# The Lever Sign Test Demonstrates Limited Clinical Utility for Diagnosing Full-Thickness Anterior Cruciate Ligament Tears After a Traumatic Knee Injury

**DOI:** 10.1177/23259671251334775

**Published:** 2025-05-14

**Authors:** Richard Norris, Alan Price, Joseph Byrne, Sian Pulford, Nicky van Melick, Thomas W. Maddox, William Boswell, Cronan Kerin, Rachel A. Oldershaw

**Affiliations:** † Department of Trauma and Orthopaedics, Aintree University Hospital, NHS University Hospitals of Liverpool Group, Liverpool, UK; ‡ Department of Musculoskeletal and Ageing Science, Institute of Life Course and Medical Sciences, Faculty of Health and Life Sciences, University of Liverpool, Liverpool, UK; § Department of Therapies, Aintree University Hospital, NHS University Hospitals of Liverpool Group, Liverpool, UK; || Sports & Orthopedics Research Center, Anna TopSupport, Eindhoven, the Netherlands; ¶ Small Animal Teaching Hospital, Institute of Infection, Veterinary and Ecological Sciences, School of Veterinary Science, University of Liverpool, Liverpool, UK; # Department of Radiology, Aintree University Hospital, NHS University Hospitals of Liverpool Group, Liverpool, UK; ** Centre for Integrated Research into Musculoskeletal Ageing, Institute of Life Course and Medical Sciences, Faculty of Health and Life Sciences, University of Liverpool, Liverpool, UK; Investigation performed at Aintree University Hospital, NHS University Hospitals of Liverpool Group, Liverpool, UK

**Keywords:** anterior cruciate ligament, lever sign test, interrater reliability, concurrent validity

## Abstract

**Background:**

Current systematic reviews with meta-analyses have identified the lever sign test as the best clinical examination for ruling out an anterior cruciate ligament (ACL) tear, but the included studies have methodological limitations that could bias the test outcome, potentially overestimating its clinical utility.

**Purpose:**

To investigate the interrater reliability and concurrent validity of the lever sign test after a traumatic knee injury and to investigate the association between test variables (surface used, fist position, effusion grade, force applied, pain reported) and test outcomes.

**Study Design:**

Cohort study (Diagnosis); Level of evidence, 2.

**Methods:**

The lever sign test was performed on hard and soft surfaces in 101 participants after a traumatic knee injury. Magnetic resonance imaging was used as the reference standard, with index testing performed after magnetic resonance imaging was conducted (>3 weeks after injury). Agreement between observers based on the surface used and fist position was evaluated with the Cohen kappa coefficient (κ). Concurrent validity was assessed through sensitivity, specificity, and likelihood ratios. Logistic regression was used to determine whether effusion grade, force applied, and pain reported were significantly associated with test outcomes.

**Results:**

Interrater reliability was superior on the soft surface but demonstrated only moderate agreement (κ = 0.529 [95% CI, 0.368-0.691]). Sensitivity was higher on the soft surface, and specificity was higher on the hard surface, for both assessors. At best, positive and negative likelihood ratios were 3.02 (95% CI, 1.60-5.69) and 0.45 (95% CI, 0.28-0.73), respectively. Test outcomes were affected by the surface used and fist position, but effusion grade, force applied, and pain reported were not significantly associated with correct/incorrect test results.

**Conclusion:**

In participants assessed from 3 weeks after a traumatic knee injury, the lever sign test demonstrated limited clinical utility for diagnosing full-thickness ACL tears. Test outcomes were affected by the surface used and fist position of the assessor.

**Registration:**

NCT05416632 (ClinicalTrials.gov)

Anterior cruciate ligament (ACL) tears are common, with an estimated 200,000 injuries occurring each year in the United States alone.^21^ Potential consequences of an ACL tear include further knee injuries, posttraumatic osteoarthritis, and reduced quality of life^13^; therefore, a prompt and accurate diagnosis is required to expedite treatment and mitigate these risks. Arthroscopic surgery is the gold standard for diagnosing ACL tears, with magnetic resonance imaging (MRI) considered the noninvasive reference standard.^33,37^ Although diagnostically accurate, MRI and arthroscopic surgery are contraindicated for certain patients, associated with known risks, high costs, and delayed diagnoses; therefore, a clinical evaluation is recommended before utilizing these advanced diagnostic techniques.^13^

ACL tears are diagnosed clinically by combining the patient's history with physical examination findings.^13^ History includes a traumatic pivoting mechanism (typically without direct contact to the knee), a “popping” or “snapping” sensation, marked intra-articular swelling, and symptomatic knee instability.^8,9,15,16,22,42,43^ Based on the most recent systematic reviews with meta-analyses investigating the concurrent validity of clinical tests, the pivot-shift and lever sign tests have demonstrated the highest diagnostic accuracy for ruling in and ruling out an ACL tear, respectively.^19,38,40^ The lever sign test, conceived in 2005, involves placing a closed fist under the proximal calf while applying moderate force to the distal thigh.^25^ One key feature of the test is that the outcome is dichotomous, depending on whether the heel rises off the examination table.^25^ Although the lever sign test has also been shown to demonstrate almost perfect interrater reliability,^26^ it has been suggested that the methodological quality of previous studies could be compromised by numerous factors that may bias the test outcome, potentially overestimating its clinical utility.^18,34^ Furthermore, previous studies have not been reported using the Guidelines for Reporting Reliability and Agreement Studies (GRRAS)^23^ or the Standards for Reporting of Diagnostic Accuracy Studies (STARD),^7^ which hinders the assessment of the risk of bias and limits the applicability of the findings.^7^

Overestimation of a test's clinical utility can negatively affect patient care, highlighting the importance of determining accurate reliability and validity estimates for the lever sign test. The primary objective of this study was to investigate the interrater reliability and concurrent validity of the lever sign test for diagnosing full-thickness ACL tears after a traumatic knee injury using recommended guidelines. The lever sign test was performed on hard and soft surfaces, as different surfaces are thought to influence outcomes.^6,28^ Testing was conducted by 2 physical therapists, with one assessor blinded to all clinical information and reference standard results. The first hypothesis was that reliability and validity would be superior on the hard surface. The second hypothesis was that validity estimates would be inferior to those reported in a recent bivariate meta-analysis (sensitivity: 0.83; specificity: 0.91; positive likelihood ratio [LR+]: 9.66; negative likelihood ratio [LR–]: 0.18).^38^ The secondary objective was to investigate the association between test variables (effusion grade, fist position, force applied, surface used, and pain reported) and test outcomes.

## Methods

### Study Design

This was a prospective cross-sectional study using a within-participant repeated-measures design that was approved by ethics committees. Signaling questions from the Consensus-based Standards for the Selection of Health Measurement Instruments (COSMIN)^30^ and Quality Assessment of Diagnostic Accuracy Studies (QUADAS-2)^44^ risk of bias tools were taken into consideration when designing the study to mitigate bias. The study was registered a priori at ClinicalTrials.gov (NCT05416632) and conducted in accordance with the ethical standards of the World Medical Association's Declaration of Helsinki (2002). The study is reported using the GRRAS^23^ and STARD^7^ guidelines as well as the Checklist for Statistical Assessment of Medical Papers (CHAMP).^27^

### Participants

#### Sample Size

For the assessment of interrater reliability, with an expected Cohen kappa coefficient (κ) of 0.80,^26^ precision of 0.15, proportion of outcomes of 50%,^32^ and attrition rate of 10%, a minimum sample size of 74 was calculated. For the assessment of validity, with an anticipated sensitivity and specificity of 0.83 and 0.91, respectively,^38^ a minimum sample size of 85 was calculated. To meet the COSMIN recommendations for an “excellent” sample size for reliability, a sample ≥100 is suggested^41^; because this yielded the largest sample size, the sample was increased to a minimum of 100 participants.

#### Study Population

To ensure that the population of interest was as similar as possible to that in which the test would be applied in practice, patients presenting to an outpatient acute knee injury clinic (AKIC) at Aintree University Hospital were used as the study population. Patients are referred to the AKIC from the accident and emergency department (AED) after a traumatic soft tissue knee injury. AKIC patients are managed according to their individual presentations and referred for MRI based on the hospital's clinical pathway (ie, joint instability, suspected traumatic meniscal tear, extension deficit/locked knee).

#### Inclusion and Exclusion Criteria

Consecutive patients returning to the AKIC who had undergone MRI were approached by direct invitation and deemed eligible for inclusion if they were aged ≥18 years and willing and able to give informed consent. Patients with MRI-confirmed partial ACL tears were not included in the study, as the pathomechanics of the lever sign test has not been validated,^20^ and it is unclear whether partial tears are expected to produce a positive or negative outcome. Patients with fractures were only included if the fracture did not affect management (eg, Segond fracture), as determined by a consulting orthopaedic surgeon. Other than standard acute knee injury management,^12^ no interventions were performed between MRI and index testing unless indicated (eg, bracing for an MRI-diagnosed complete ligament tear). Index testing was conducted in the outpatient physical therapy department before commencing further assessments and treatment. Participants with a healthy ACL on MRI who reported an additional injury between MRI and the index testing session were excluded, unless further MRI was performed to re-evaluate the integrity of the ACL.

### Imaging

MRI was used as the reference standard, as it is likely to correctly classify the target condition,^3,33,37^ and the routine use of arthroscopic surgery is not appropriate after traumatic knee injuries.^35^ All MRI examinations were performed using a 1.5- or 3.0-T system (Ingenia; Philips), with results reported by a consulting musculoskeletal radiologist. For patients who underwent surgery during the study period, arthroscopic diagnoses were used to determine whether MRI had correctly classified the integrity of the ACL but were not included in analyses.

### Testing

#### Timing and Flow

The lever sign test was performed as soon as possible after MRI to avoid potential changes in a participant's status. For example, ACL healing may be evident on MRI as early as 3 months after an injury,^14^ which could potentially affect the test outcome. Participants with a healthy ACL on imaging were questioned directly during the index testing session to confirm that they had not reinjured their knee since MRI. The same reference standard was used for all participants, and all participant data were included in analyses.

#### Assessors

The blinded assessor was a physical therapist with 8 years’ clinical experience and 8 years’ experience using the lever sign test. The unblinded assessor was a physical therapist with 21 years’ clinical experience and 9 years’ experience using the test. Both assessors have a special interest in the assessment and management of soft tissue knee injuries; the unblinded assessor has worked in an AKIC for 19 years. The width of each assessor's middle 3 fingers and closed fist was measured using a digital caliper (Kynup; Deqing Liangfeng Electronic & Technology). The unblinded assessor's middle 3 fingers (right: 55.2 mm; left: 53.4 mm) and fist (right: 84.6 mm; left: 84.6 mm) were wider than the blinded assessor's middle 3 fingers (right: 53.8 mm; left: 51.8 mm) and fist (right: 79.0 mm; left: 76.0 mm). Both assessors were right-hand dominant.

#### Procedure

**Figure 1.**
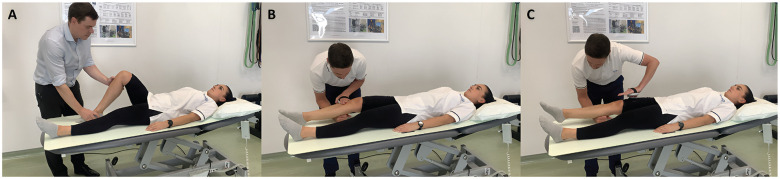
Representative images of the lever sign test performed on a hard surface with a negative test outcome. (A) Palpation of the tibial tuberosity, (B) initial fist position, and (C) force recorded.

The participants were asked to lie supine on a cushioned examination table (AKRON) with their footwear removed. Before testing, the unblinded assessor examined the knee to ensure that there was sufficient range of motion to perform the lever sign test^36^ and to grade knee effusion using the stroke test.^39^ This assessor then palpated (Figure 1A) and marked the tibial tuberosity with a surgical skin marker (Becton Dickinson); there was no disagreement between assessors regarding the location of the tibial tuberosity.

During testing, the assessor placed a closed fist under the proximal third of the calf, 3 fingers distal to the marked tibial tuberosity (Figure 1B), as described by the originator of the lever sign test (https://www.youtube.com/watch?v=eEhpwTU3KXg). Care was taken to ensure that the fist was positioned accurately relative to the marked tibial tuberosity, as fist positioning has been reported to affect test outcomes.^4^ With the participant as relaxed as possible, the assessor applied moderate downward force to the distal third of the quadriceps with the other hand^25^; a microFET2 dynamometer with a curved attachment (Hoggan Scientific) was used to quantify the force applied (in N) (Figure 1C). The lever sign test result was recorded as negative (healthy ACL) if the heel rose up off the surface (Figure 1C) and positive (torn ACL) if it did not.^25^

To determine the influence of fist positioning, the test was repeated with the fist relocated, depending on the outcome of the initial test. If the initial test result was negative, the fist was moved proximally in line with the tibial tuberosity (Supplemental Video), but if the initial test result was positive, the fist was moved 3 fingers distal to the starting position. This was based on observations during pilot testing that proximal and distal fist positions were associated with more positive and negative results, respectively. Retest outcomes were used to determine the agreement between each assessor's outcomes based on fist positioning but were not included in interrater reliability or validity analyses.

Testing was performed on soft and hard surfaces (cushioned examination table with and without a 1525-mm × 635-mm Patslide [Briggate Medical Company]), with all participants evaluated on both surfaces. After each test, the participant was asked to report any pain experienced during testing on a numerical rating scale (0-10, with 0 representing no pain and 10 representing the worst pain imaginable). Each assessor recorded any tests that they thought were affected by participant guarding.

#### Randomization

The order of testing for assessors and surfaces was randomized to control for potential sources of systematic errors including participant guarding or relaxation from repeated testing using an online random item generator (www.random.org/lists). All tests were performed during the same assessment session with no intervals between tests.

#### Blinding

The blinded assessor performed and interpreted the lever sign test without knowledge of any clinical information or MRI results and was not allowed to talk to the participants other than to explain what they were doing. To determine whether blinding was successfully achieved, the blinded assessor was asked to make a diagnosis (healthy ACL or torn ACL) based on any information gathered during testing. To reflect normal clinical practice, the unblinded assessor had access to clinical history, other examination findings, and imaging results. Assessors were blinded to their own force values and each other's outcomes until testing was complete, and all MRI scans were interpreted by the consulting radiologist without knowledge of the index test results. Because of the requirements for informed consent, patients were not blinded to their MRI results, were told that the lever sign test would be performed, but were not informed about what constitutes a positive or negative test outcome. The lever sign test was not performed on any participant before data collection.

### Statistical Analysis

All statistical analyses were performed using SPSS software (Version 29.0; IBM), and all relevant assumptions for the statistical tests were considered. Continuous variables were assessed for normality by graphical analysis (histograms and normal Q-Q plots) and normality testing (Kolmogorov-Smirnov and Shapiro-Wilk tests). Point estimates for statistical tests are reported with 95% confidence intervals (CIs) where appropriate.

Interrater reliability and agreement between each assessor's outcomes (ie, hard vs soft surface and initial fist position vs repositioned fist) were evaluated with the κ value. The strength of agreement was categorized as disagreement <0.00, poor between 0.00-0.20, fair between 0.21-0.40, moderate between 0.41-0.60, good between 0.61-0.80, and very good between 0.81-1.00.^1^

For concurrent validity, contingency tables (number of true positives, false positives, true negatives, and false negatives) were constructed by cross-tabulating the index test and MRI outcomes. For each assessor and surface, sensitivity, specificity, likelihood ratios, predictive values, accuracy, and diagnostic odds ratio were calculated. In the context of this study, the likelihood ratios indicate how likely the lever sign test result will be positive or negative, depending on the ACL status. For example, an LR+ of 5 suggests a patient with a full-thickness ACL tear is 5 times more likely to have a positive result than someone with a healthy ACL, whereas an LR– of 0.2 suggests a person with a healthy ACL is 5 times more likely to have a negative result than a patient with a full-thickness ACL tear. For LR+, the shift in pretest to posttest probability was categorized as no change if 1, minimal if 1-2, small if 2-5, moderate if 5-10, and large if >10. For LR–, the shift was categorized as no change if 1.0, minimal if 1.0-0.5, small if 0.5-0.2, moderate if 0.2-0.1, and large if <0.1. Fagan nomograms were used to illustrate the shift in the pretest to posttest probability of diagnosing a full-thickness ACL tear based on a positive or negative lever sign test result.

The paired-samples *t* test and Wilcoxon signed-rank test were performed to determine whether there was a significant difference in the mean force applied or median pain reported, respectively, between assessors and surfaces. The independent-samples *t* test and Mann-Whitney *U* test were used to determine whether there was a significant difference in the mean force applied and median pain reported, respectively, between groups (torn ACL or healthy ACL).

Binomial logistic regression was performed to determine if there was a statistically significant association between independent test variables (effusion grade, force applied, pain reported) and positive or negative test outcomes. Additional logistic regression was performed to determine the association between the same independent test variables and correct (true positives and true negatives) or incorrect (false positives and false negatives) test outcomes. Separate logistic regression models were used for each assessor and surface. The linearity of the continuous variables with respect to the logit of the dependent variable was evaluated via the Box-Tidwell method.^2^

## Results

**Figure 2.**
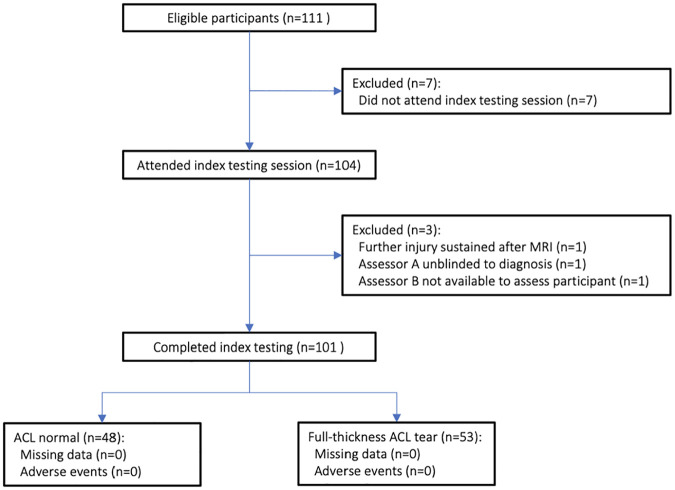
Flow of eligible participants from recruitment to the index testing session. MRI, magnetic resonance imaging.

A total of 111 participants were recruited for the study, of whom 10 were excluded (Figure 2). Of the 101 included participants, 53 (52.5%) had an MRI-diagnosed full-thickness ACL tear (35 male, 18 female), and 48 (47.5%) had a healthy ACL (34 male, 14 female). No adverse events occurred during MRI or index testing, and no data were missing.

There was no significant difference in demographic characteristics or time intervals between groups, except for the number of days from the AKIC to MRI (Table 1). Overall, 2 participants (2.0%) were deemed to be guarding by the unblinded assessor and 1 (1.0%) by the blinded assessor. The blinded assessor correctly guessed 53.8% of the diagnoses, indicating successful blinding. Additionally, 6 participants (5.9%) underwent arthroscopic surgery during the study period. Of these, MRI had correctly classified the integrity of all ACLs (100.0%).

**Table 1:** Participant Characteristics and Time Between Assessments^*a*^

	Healthy ACL (n = 48)		Torn ACL (n = 53)		*P*
	Mean ± SD	Median (IQR)	Mean ± SD	Median (IQR)	
Age, y	27.0 ± 8.9	23.0 (21.0 – 31.3)	27.2 ± 9.1	24.0 (21.0 – 30.0)	.867
Weight, kg	84.8 ± 18.6	84.1 (69.3 – 93.4)	84.4 ± 16.5	84.3 (72.1 – 96.9)	.919
Height, m	1.79 ± 0.09	1.80 (1.73 – 1.87)	1.75 ± 0.10	1.76 (1.67 – 1.81)	.070
Body mass index, kg/m^2^	26.6 ± 6.3	25.3 (22.3 – 28.5)	26.0 ± 4.4	26.2 (23.8 – 29.7)	.919
Injury to AKIC, d	15.6 ± 10.9	14.0 (8.8 – 18.3)	16.3 ± 11.4	11.0 (8.0 – 19.0)	.943
AKIC to MRI, d	45.6 ± 38.9	34.0 (25.8 – 47.8)	32.6 ± 27.6	25.0 (16.0 – 39.0)	.019
MRI to index test, d	53.4 ± 47.1	38.0 (26.8 – 64.5)	43.1 ± 36.8	35.0 (19.5 – 54.5)	.233
Injury to index test, d	99.0 ± 65.9	77.0 (59.3 – 105.0)	83.6 ± 42.2	72.0 (55.0 – 105.0)	.546

a *P* values for weight and height were derived from the independent *t* test. *P* values for age, body mass index, and time intervals were derived from the Mann-Whitney *U* test. ACL, anterior cruciate ligament; AKIC, acute knee injury clinic; IQR, interquartile range; MRI, magnetic resonance imaging.

### Interrater Reliability and Agreement

Point estimates for interrater reliability were fair on the hard surface (κ = 0.258 [95% CI, 0.071-0.445]) and moderate on the soft surface (κ = 0.529 [95% CI, 0.368-0.691]). Agreement between each assessor's outcomes on the hard and soft surfaces was moderate for the blinded assessor (κ = 0.418 [95% CI, 0.256-0.580]) and unblinded assessor (κ = 0.594 [95% CI, 0.448-0.740]).

There was disagreement (κ < 0.00) between outcomes for the initial and repositioned fist locations for the blinded assessor (hard surface: κ = −0.019 [95% CI, –0.195 to 0.157]; soft surface: κ = −0.066 [95% CI, –0.237 to 0.105]) and unblinded assessor (hard surface: κ = −0.405 [95% CI, –0.580 to −0.229]; soft surface: κ = −0.510 [95% CI, –0.667 to −0.352]). For the blinded assessor, 47 outcomes changed after repositioning the fist on the hard surface (46.5%), and 56 changed on the soft surface (55.4%). For the unblinded assessor, 82 outcomes changed on the hard surface (81.2%), and 76 changed on the soft surface (75.2%).

### Concurrent Validity

**Figure 3.**
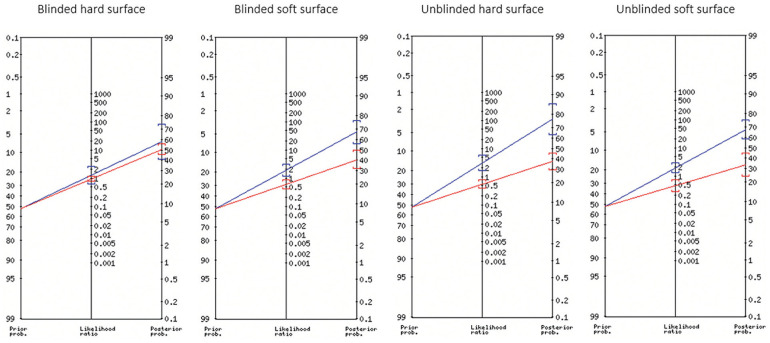
Fagan nomograms illustrating the shift in the pretest to posttest probability of diagnosing a full-thickness anterior cruciate ligament (ACL) tear for each assessor and surface. The pretest probability was 52.5% (prevalence of full-thickness ACL tears in this study population), with the posttest probability indicated by the blue (positive lever sign test outcome) and red (negative lever sign test outcome) lines.

For both assessors, sensitivity was higher on the soft surface, and specificity was higher on the hard surface (Table 2 and Appendix Section 1). For the blinded assessor, LR+ and LR– point estimates produced minimal shifts in pretest to posttest probability on both surfaces (Table 2). For the unblinded assessor, the LR+ and LR– produced small shifts on the hard and soft surfaces (Table 2). For the blinded assessor on the hard surface, the probability of a full-thickness ACL tear was 58% (95% CI, 41%-74%) with a positive result and 51% (95% CI, 45%-56%) with a negative result; on the soft surface, the posttest probability was 67% (95% CI, 56%-77%) and 40% (95% CI, 32%-49%), respectively (Figure 3).

**Table 2:** Concurrent Validity Estimates for Lever Sign Test^*a*^

	Hard Surface		Soft Surface	
	Blinded Assessor	Unblinded Assessor	Blinded Assessor	Unblinded Assessor
Sensitivity	0.26 (0.15-0.40)	0.57 (0.42-0.70)	0.58 (0.44-0.72)	0.72 (0.58-0.83)
Specificity	0.79 (0.65-0.90)	0.81 (0.67-0.91)	0.69 (0.54-0.81)	0.63 (0.47-0.76)
LR+	1.27 (0.62-2.58)	3.02 (1.60-5.69)	1.87 (1.16-3.02)	1.91 (1.28-2.86)
LR–	0.93 (0.75-1.15)	0.53 (0.38-0.75)	0.60 (0.42-0.88)	0.45 (0.28-0.73)
PPV	0.58 (0.37-0.78)	0.77 (0.61-0.89)	0.67 (0.52-0.80)	0.68 (0.54-0.80)
NPV	0.49 (0.38-0.61)	0.63 (0.50-0.75)	0.60 (0.46-0.73)	0.67 (0.51-0.80)
Accuracy	0.51 (0.41-0.62)	0.68 (0.58-0.77)	0.63 (0.53-0.73)	0.67 (0.57-0.76)
DOR	1.36 (0.54-3.44)	5.65 (2.28-13.98)	3.10 (1.37-7.03)	4.22 (1.83-9.74)

a 95% CIs are in parentheses. DOR, diagnostic odds ratio; LR–, negative likelihood ratio; LR+, positive likelihood ratio; NPV, negative predictive value; PPV, positive predictive value.

### Effusion Grade

Of the 53 participants with an ACL tear, 20 had grade 0 effusion (37.7%), 5 were recorded as trace (9.4%), 18 grade 1+ (34.0%), 9 grade 2+ (17.0%), and 1 grade 3+ (1.9%). For the 48 participants with a healthy ACL, 33 had grade 0 effusion (68.8%), 4 were recorded as trace (8.3%), 7 grade 1+ (14.6%), 2 grade 2+ (4.2%), and 2 grade 3+ (4.2%).

### Force Applied

Both assessors applied significantly higher mean force on the soft surface than on the hard surface (Table 3), with the unblinded assessor applying significantly higher mean force than the blinded assessor on both surfaces (Table 4). For positive and negative lever sign test outcomes, the assumption of the homogeneity of variance was violated for the blinded assessor on the soft surface and the unblinded assessor on the hard surface (Levene test = 0.002 and 0.012, respectively); therefore, the Welch *t* test was performed for these data. Both assessors applied significantly higher mean force in participants with positive outcomes than in those with negative outcomes (Table 5). There was no significant difference in mean force applied in participants with healthy or torn ACLs for either assessor (Table 6).

**Table 3:** Paired-samples *t* Test Results for Force Applied Between Surfaces^*a*^

Assessor	Force Applied, Mean ± SD, N		Difference, Mean (95% CI), N	*t*	df	*P*	Cohen *d*
	Soft Surface	Hard Surface					
Blinded	166.7 ± 47.0	149.0 ± 50.8	17.7 (11.4-23.9)	5.595	100	<.001	0.557
Unblinded	175.6 ± 41.5	164.7 ± 50.4	10.9 (4.0-17.8)	3.151	100	.002	0.314

a df, degrees of freedom.

**Table 4:** Paired-samples *t* Test Results for Force Applied Between Assessors^*a*^

Surface	Force Applied, Mean ± SD, N		Difference, Mean (95% CI), N	*t*	df	*P*	Cohen *d*
	Unblinded Assessor	Blinded Assessor					
Soft	175.6 ± 41.5	166.7 ± 47.0	8.9 (0.1-17.7)	2.010	100	.047	0.200
Hard	164.7 ± 50.4	149.0 ± 50.8	15.7 (6.0-25.4)	3.196	100	.002	0.318

a df, degrees of freedom.

**Table 5:** Independent-samples *t* Test Results for Force Applied Between Participants With Positive or Negative Outcomes^*a*^

Assessor (Surface)	Force Applied, Mean ± SD, N		Difference, Mean (95% CI), N	*t*	df	*P*	Cohen *d*
	Positive Outcome	Negative Outcome					
Blinded (soft)	193.2 ± 30.4	144.5 ± 47.2	48.7 (33.2-64.1)	6.253	93.262	<.001	1.204
Blinded (hard)	187.1 ± 33.6	137.2 ± 49.5	49.9 (28.5-71.4)	4.618	99	<.001	1.079
Unblinded (soft)	188.7 ± 34.4	159.3 ± 44.1	29.4 (13.9-44.9)	3.763	99	<.001	0.753
Unblinded (hard)	197.1 ± 34.9	144.4 ± 48.1	52.7 (36.2-69.1)	6.489	96.864	<.001	1.211

a df, degrees of freedom.

**Table 6:** Independent-samples *t* Test Results for Force Applied Between Participants With Healthy or Torn ACLs^*a*^

Assessor (Surface)	Force Applied, Mean ± SD, N		Difference, Mean (95% CI), N	*t*	df	*P*	Cohen *d*
	Healthy ACL	Torn ACL					
Blinded (soft)	169.4 ± 51.9	163.7 ± 41.4	5.7 (–13.0 to 24.3)	0.603	99	.548	0.120
Blinded (hard)	149.2 ± 55.2	148.8 ± 45.9	0.4 (–19.8 to 20.6)	0.039	99	.969	0.008
Unblinded (soft)	178.7 ± 43.2	172.3 ± 39.8	6.4 (–10.0 to 22.9)	0.777	99	.439	0.155
Unblinded (hard)	171.1 ± 54.6	157.7 ± 44.7	13.5 (–6.4 to 33.3)	1.347	99	.181	0.268

a ACL, anterior cruciate ligament; df, degrees of freedom.

### Pain Reported

The median pain reported was 0.0 on the soft surface (interquartile range [IQR], 0.0-3.0) and hard surface (IQR, 0.0-2.0) for the blinded assessor and 1.0 on the soft surface (IQR, 0.0-3.0) and hard surface (IQR, 0.0-3.0) for the unblinded assessor. Participants reported significantly lower median pain for the blinded assessor than for the unblinded assessor on the soft surface (*Z* = −2.660; *P* = .008) and hard surface (*Z* = −3.259; *P* = .001). The median pain reported was significantly lower on the hard surface than on the soft surface for the blinded assessor (*Z* = −2.620; *P* = .009) and unblinded assessor (*Z* = −1.839; *P* = .066). The median pain reported was significantly lower in participants with healthy ACLs than in those with torn ACLs for the blinded assessor on the soft surface (*U =* 945; *Z* = −2.399; *P* = .016) and hard surface (*U =* 979; *Z* = −2.205; *P* = .027) and for the unblinded assessor on the soft surface (*U =* 893; *Z* = −2.709; *P* = .007) and hard surface (*U =* 921; *Z* = −2.533; *P* = .011).

### Binomial Logistic Regression

All continuous independent variables were found to be linearly related to the logit of the dependent variable. There were 7 standardized residuals with a value >2.5 standard deviations, which were inspected and included in analyses.

The logistic regression models for the probability of a positive/negative lever sign test result based on effusion grade, force applied, and pain reported were statistically significant for the blinded assessor on the soft surface (χ^2^(12) = 47.854; *P* < .001) and hard surface (χ^2^(12) = 48.670; *P* < .001) and for the unblinded assessor on the soft surface (χ^2^(13) = 33.482; *P* = .001) and hard surface (χ^2^(12) = 59.261; *P* < .001). Of the 3 predictor variables, only force applied was statistically significant (*P* < .001) for both assessors on both surfaces (Appendix Section 2).

The logistic regression models for the probability of a correct/incorrect lever sign test result based on effusion grade, force applied, and pain reported were not statistically significant for the blinded assessor on the soft surface (χ^2^(10) = 15.781; *P* = .106) and hard surface (χ^2^(11) = 11.599; *P* = .395) or for the unblinded assessor on the soft surface (χ^2^(12) = 19.137; *P* = .085) and hard surface (χ^2^(11) = 16.364; *P* = .128).

## Discussion

The most important findings from the current study are that the lever sign test demonstrated comparable concurrent validity between hard and soft surfaces but lower interrater reliability on the hard surface; therefore, the first hypothesis is rejected. Regardless of the surface used, validity estimates were inferior to those reported in a recent bivariate meta-analysis; therefore, the second hypothesis is accepted. The lever sign test outcomes were affected by the surface used and fist position, but effusion grade and pain reported were not significantly associated with positive/negative or correct/incorrect test outcomes. Force applied was significantly associated with positive/negative test outcomes but not correct/incorrect test outcomes. Based on the findings from this study, the lever sign test demonstrated limited clinical utility for diagnosing full-thickness ACL tears after a traumatic knee injury, particularly when performed on a hard surface.

### Reliability

An essential requirement of all measurements in clinical practice is that they are reliable.^11^ Reliability is a characteristic of a test used in a population, not simply the test itself, and should be investigated in a sample representative of that in which the test is to be used.^11^ ACL tears are caused by a traumatic injury to the knee,^8^ and the interrater reliability of the lever sign test was therefore investigated within this context. Previous studies have included patients with no history of injuries^24^ or those requiring surgery^6,10,26^ and are at risk of selection bias.

In the current study, interrater reliability was fair on the hard surface, which is consistent with point estimates reported previously^6^ and reinforces the issues with reliability on this surface. Lichtenberg et al^26^ reported almost perfect interrater reliability (κ = 0.82), but the surface used was not described, and it is not clear whether assessors were blinded to clinical information that could bias the test outcome. Deveci et al^10^ reported interrater reliability estimates of 0.89 to 0.96 using the intraclass correlation coefficient, but this parameter of reliability is not appropriate for dichotomous outcomes.^11^

### Validity

An ideal diagnostic test should not produce any false-positive or false-negative results.^5^ The lever sign test was originally investigated in 400 participants with MRI-diagnosed partial or complete ACL tears using the contralateral limb as a healthy control.^25^ No false-positive or false-negative outcomes were recorded, producing perfect sensitivity and specificity values. In the first systematic review with meta-analysis to include the lever sign test,^34^ the pooled results were found to be heavily influenced by the original study, indicating confirmation bias. Although updated meta-analyses have reported more realistic estimates for concurrent validity, the included studies demonstrated limited quality with a high risk of bias.^18^ Several studies also reported wide 95% CIs for sensitivity or specificity because of the disproportionate number of ACL tears^4,28^ or controls^20,29^ recruited.

In the current study, 52.5% of participants had an MRI-diagnosed ACL tear, which is consistent with previous data reported for patients presenting to an AED with knee hemarthrosis after a traumatic injury.^32^ Both assessors recorded more negative outcomes on the hard surface and more positive outcomes on the soft surface presumably because of the reduced action of the fulcrum as the assessor's fist sank into the softer surface. Accordingly, there were more false negatives on the hard surface and more false positives on the soft surface, which is in contrast to previous unvalidated claims that soft surfaces are associated with false negatives.^6^ The LR− was higher for both assessors on the hard surface, and the LR+ was lower for the blinded assessor on the hard surface, contradicting previous assumptions that the test has better performance on this surface.^6,28^ Although the LR+ was higher on the hard surface for the unblinded assessor, this produced only a small shift in the pretest (52.5%) to posttest (77.0%) probability of diagnosing an ACL tear (Figure 3).

### Testing Procedure

The lever sign test is a quick and simple examination that requires minimal equipment but appears susceptible to numerous external factors that can influence the outcome. In the current study, 64.6% of outcomes could be changed by moving the fist approximately 55 mm, which is relevant because previous studies have inadequately described the fist location under the calf^4,6,10,17,20,25,26,31,36^ or have used more proximal fist positioning.^28,29^ Because the test utilizes a first-class lever system, moving the fulcrum will simultaneously affect the downward torque exerted by the assessor on the femur and the mass of the leg distal to the fulcrum.

The position of the force-applying hand was not standardized in the current study, as there is no specific guidance on where this should be placed. Most studies have used the distal third of the quadriceps, with one study placing the hand 10 cm proximal to the patella without clarifying whether this was measured.^28^ Moderate downward force is applied by this hand^25^; 30 lb (133 N) of force on a hard surface has been proposed previously without being quantified.^31^ Both assessors applied higher mean force in the current study, but the magnitude of force was not significantly associated with a correct/incorrect lever sign test outcome. Although force applied was significantly associated with a positive/negative lever sign test result, it is more likely that the outcome dictates the force applied rather than the force affecting the outcome. Once the heel lifts (negative outcome), the assessor will stop pushing, but if the heel does not lift (positive outcome), the assessor will apply more force, as indicated by the significantly higher mean force applied by both assessors in participants with positive outcomes.

### Pain

One proposed advantage of the lever sign test is that it is less painful than other clinical tests.^19^ Pain reported ranged from 0 to 9 of 10, which is consistent with previous observations that the test can be unpleasant or painful.^4^ Despite this, pain reported was not significantly associated with lever sign test outcomes in the logistic regression models.

### Strengths, Limitations, and Recommendations

This is the first study on the lever sign test to be reported in accordance with the GRRAS, STARD, and CHAMP guidelines. Signaling questions from the COSMIN and QUADAS-2 risk of bias tools were used for the study design to mitigate bias, and the sample size is considered excellent. This is also the first study to quantify the force applied during the lever sign test and to investigate the association between the surface used, effusion grade, fist position, force applied, and pain reported on test outcomes.

All participants were referred from an AED; thus, the findings may not be generalizable to patients presenting to non–emergency departments. Index testing was not performed within 3 weeks of the injury because of hospital wait times for MRI and clinical reviews; therefore, the results may not be generalizable to patients presenting sooner. A total of 13 (24.5%) index tests were performed in participants with ACL tears more than 3 months after the injury, and a proportion of these tears could have healed. In that scenario, the number of false negatives would decrease, and the number of false positives would increase, resulting in higher sensitivity and lower specificity values, respectively, but similar likelihood ratios.

MRI was deemed to be indicated for participants recruited to the study, which may introduce selection bias, but MRI is recommended to confirm a suspected ACL tear and to assess for concomitant injuries.^13^ The diagnostic accuracy of MRI is dependent on magnetic field strength,^33,37^ and arthroscopic surgery remains the gold standard; therefore, results should be interpreted accordingly.

Partial ACL tears were excluded from the study, as it is unclear whether these injuries would produce positive or negative test results. Cadaveric studies could be conducted to investigate the mechanics of the lever sign test before and after ACL sectioning, accounting for the fulcrum position and the magnitude of force applied. Although there was a negligible difference in hand size between assessors in the current study, a more consistent approach would be to position a standardized fulcrum (eg, dense foam pad) at a set distance from an anatomic landmark using a tape measure. The downward force applied could also be standardized using dynamometry.

## Conclusion

In participants assessed at least 3 weeks after a traumatic knee injury, the lever sign test demonstrated limited clinical utility for diagnosing full-thickness ACL tears. Concurrent validity estimates for the lever sign test were comparable between hard and soft surfaces, but interrater reliability was superior on the soft surface. Test outcomes were influenced by the surface used and fist position, but effusion grade, force applied, and pain reported were not significantly associated with correct/incorrect lever sign test outcomes.
